# Molecular Study of Thyroid Cancer in World Trade Center Responders

**DOI:** 10.3390/ijerph16091600

**Published:** 2019-05-07

**Authors:** Maaike A. G. van Gerwen, Stephanie Tuminello, Gregory J. Riggins, Thais B. Mendes, Michael Donovan, Emma K.T. Benn, Eric Genden, Janete M. Cerutti, Emanuela Taioli

**Affiliations:** 1 Institute for Translational Epidemiology and Department of Population Health Science and Policy, Icahn School of Medicine at Mount Sinai, New York, NY 10029, USA; maaike.vangerwen@icahn.mssm.edu (M.A.G.v.G.); stephanie.tuminello@mssm.edu (S.T.); 2 Department of Neurosurgery, Johns Hopkins University School of Medicine, Baltimore, MD 21218, USA; griggin1@jhmi.edu; 3 Division of Genetics, Universidade Federal de São Paulo, São Paulo 04039-032, Brazil; thais_biude@hotmail.com (T.B.M.); j.cerutti@unifesp.br (J.M.C.); 4 Department of Pathology, Icahn School of Medicine at Mount Sinai, New York, NY 10029, USA; michael.donovan@mssm.edu; 5 Department of Population Health Science and Policy, Center for Biostatistics, Icahn School of Medicine at Mount Sinai, New York, NY 10029, USA; emma.benn@mountsinai.org; 6 Department of Otolaryngology- Head and Neck Surgery, Icahn School of Medicine at Mount Sinai, New York, NY 10029, USA; Eric.Genden@mountsinai.org; 7 Department of Thoracic Surgery, Icahn School of Medicine at Mount Sinai, New York, NY 10029, USA; 8 Tisch Cancer Institute, Icahn School of Medicine at Mount Sinai, New York, NY 10029, USA

**Keywords:** 9/11, screening, thyroid cancer, biomarkers

## Abstract

Thyroid cancer incidence is higher in World Trade Center (WTC) responders compared with the general population. It is unclear whether this excess in thyroid cancer is associated with WTC-related exposures or if instead there is an over-diagnosis of malignant thyroid cancer among WTC first responders due to enhanced surveillance and physician bias. To maximize diagnostic yield and determine the false positive rate for malignancy, the histological diagnoses of thyroid cancer tumors from WTC responders and age, gender, and histology matched non-WTC thyroid cancer cases were evaluated using biomarkers of malignancy. Using a highly accurate panel of four biomarkers that are able to distinguish benign from malignant thyroid cancer, our results suggest that over-diagnosis by virtue of misdiagnosis of a benign tumor as malignant does not explain the increased incidence of thyroid cancer observed in WTC responders. Therefore, rather than over-diagnosis due to physician bias, the yearly screening visits by the World Trade Center Health Program are identifying true cases of thyroid cancer. Continuing regular screening of this cohort is thus warranted.

## 1. Introduction

A significantly increased risk of thyroid cancer has been reported in the Mount Sinai Health Program of World Trade Center (WTC) responders [1], WTC-exposed firefighters [2], and the New York City Department of Health exposed residents [3], with an excess risk in the range of 2–3 times the incidence reported by cancer registries.

The etiology behind this increased incidence of thyroid cancer remains unclear. Though multiple carcinogens were identified at Ground Zero, including soot, benzene, and other volatile organic compounds from the burning of the jet fuel, in addition to the asbestos, silica, and other fibers that were in the dust caused by the towers collapse, none are known to act as carcinogens on the thyroid [4]. Established risk factors for thyroid cancer, such as exposure to radiation or iodine-131, have not been reported in connection to Ground Zero [5].

Instead, overdiagnosis as a consequence of surveillance and physician biases has been suggested as a possible explanation of the excess risk of thyroid cancer in the WTC cohort [1]. Enhanced surveillance leading to heightened diagnosis is a well-recognized phenomenon in heavily screened populations [6]. This phenomenon has been suggested as an explanation for other cancers for which WTC responders have been observed to be at increased risk, such as prostate cancer [7]. Funded under the James Zadroga 9/11 Health and Compensation Act of 2010, the World Trade Center Health Program (WTCHP) monitors the health of WTC responders with yearly visits, including a physical examination, laboratory testing, spirometry, and chest radiography. Chest imaging is known to increase detection of incidental thyroid nodules [8], and WTC responders, many of whom report respiratory health problems [9], are referred for imaging at higher rates, thus making the chance of discovery of a thyroid nodule more likely. It is possible that some of these nodules would never have become clinically evident, and so would have never been discovered, if not for this heightened screening [7].

Physician’s cognitive biases are known to lead to diagnostic inaccuracies [10]. Physicians treating WTC responders, including pathologists, may suspect a worse diagnosis for these patients compared with non-WTC responders due to implicit biases associated with knowledge of the patient’s past WTC exposure. In addition, they may prefer risk aversion by a defensive diagnosing strategy to avoid missing any malignancies and classify a suspicious nodule as cancerous [10]. This, along with the increase in nodule detection due to enhanced screening through the WTCHP, may be leading to an over-diagnosis of malignant thyroid cancer among WTC first responders.

The goal of this study is to analyze pathologists’ histological diagnosis of thyroid cancer in WTC first responders and, by doing so, determine the false positive rate for malignancy. We will examine the clinical characteristics and results of a cancer-detection four-biomarker panel of WTC tumors compared with non-WTC exposed age-, gender-, and histology-matched cases.

## 2. Methods

### 2.1. Selection and Enrollment of Study Participants 

Eligible WTC responders who participated (as employees or volunteers) in rescue, recovery, and cleanup efforts at the WTC sites and who met established eligibility criteria [11] have been enrolled in the WTCHP at Mount Sinai. Over 27,000 responders have had at least one monitoring visit in the WTCHP and have consented to the aggregation of their data;20,984 of which have consented to have their records used for medical research [12].

Cancer cases are identified through periodical linkage with the cancer registries of New York, New Jersey, Pennsylvania, and Connecticut, as these states account for 98% of the responders’ residencies at time of WTCHP enrollment. The full linkage methodology has been described elsewhere [1,12]. Only thyroid cancer cases validated by a cancer registry were eligible to participate in this study. Additionally, WTC responders were only eligible if their enrollment in the WTCHP predated their cancer diagnosis.

The full methodology used for patient recruitment and consent has been previously described [12]. In brief, eligible participants were contacted by phone, and those interested in participating were mailed a consent form. After a patient’s consent, a cancer tissue sample was obtained from the hospital where the patient’s thyroid surgery had taken place and clinical information related to their cancer was abstracted from the pathology reports. For each patient, a pathologist reviewed the tissue blocks to assure that cancer tissue was present, and 5 unstained slides of 4 μm thickness, in addition to 1 hematoxylin and eosin (H/E) stained slide, were cut from the chosen formalin-fixed paraffin-embedded (FFPE) tumor block. WTC cases were one-to-one matched by gender, histology, and age (+/- 5 years) to thyroid cancer samples obtained from the Mount Sinai Biorepository, with no known WTC exposure. For cases with rare histology (*n* = 3), such as columnar cell papillary carcinoma, it was not possible to match by histology, and matching was limited to age and gender. After de-identification, all samples were sent to John Hopkins University for analysis. This study protocol was approved by the Icahn School of Medicine at Mount Sinai’s Institutional Review Board (IRB-17-01323).

### 2.2. Assessment of Clinical Characteristics 

Pathology reports relating to the thyroid cancer surgeries of the WTC cases and Mount Sinai non-WTC exposed controls were obtained, and data pertaining to the patient’s age, gender, tumor histology, and size was abstracted into an excel database. To ensure that the WTC thyroid cancer cases included in this study represented a random subset of the overall sample, characteristics of the study sample were compared with the overall WTCHP thyroid cancer cohort using clinical data provided by the WTCHP (Table 1).

### 2.3. Immunohistochemistry Analysis

**Figure 1:**
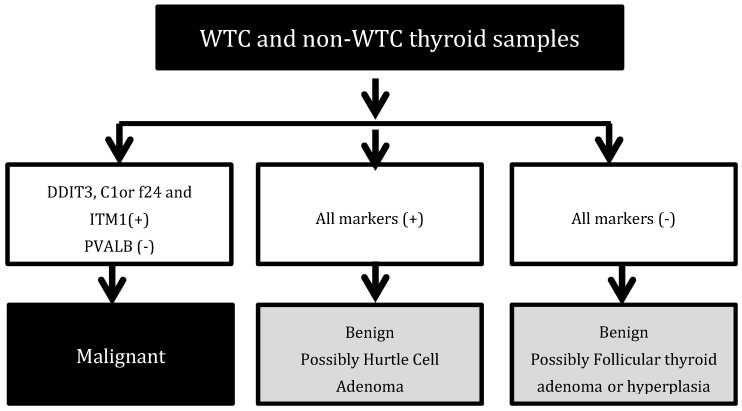
Antibody Assessment. WTC: World trade center.

While diagnosis of papillary thyroid carcinoma (PTC), the most common subtype, can usually be achieved through cytology, diagnosis of other thyroid cancer histologies can prove to be more difficult [13], resulting in benign thyroid lesions being incorrectly classified as malignant. We have previously developed a panel of molecular markers to distinguish benign from malignant thyroid cancer, which can be used to cost-effectively identify the presence of over-diagnosis [14,15,16]. As seen in Figure 1, the panel of molecular markers includes DDIT3, ITM1, C1orf24, and PVALB antibodies, the utility of which has been described elsewhere [14,15,16], which can accurately discriminate between malignant and benign tumors with a sensitivity and specificity close to 100% (Figure 1).

FFPE WTC and non-WTC thyroid tumor tissues were analyzed using these carcinoma markers. Briefly, the FFPE sections (4 μm) were deparaffinized in xylene and rehydrated through a series of graded alcohols. The endogenous alkaline phosphatase activity was blocked with 15% (vol/vol) hydrogen peroxide in deionized water for 10 minutes. To expose the PVALB protein, permeabilization was performed with 0.5% Tween in phosphate-buffered saline (PBS: 10 mM phosphate buffer, pH 7.2, containing 0.15 M NaCl) at room temperature for 20 min. For PVALB and DDIT3, antigen retrieval was performed using 0.1 M Tris-HCl for 15 min in a steamer. For C1orf24, antigen retrieval was performed in a Tris-based solution pH 7.4 (AR-10; Biogenex Laboratories, San Ramon, CA) for 15 min in a pressure cooker. For ITM1, antigen retrieval was performed using 0.1 M Tris-HCl pH 7.4 for 5 min in a pressure cooker. After antigen retrieval, the slides were allowed to cool down for 30 min. Non-specific binding sites were blocked with 5% (C1orf24) or 10% (ITM1) normal goat serum 1 h or 5% bovine serum albumin (BSA) (DDIT3 and PVALB) for 30 min prior to incubation with primary antibodies. Polyclonal anti-C1orf24 and anti-ITM1 (custom produced) were used at 1:200 dilution. Monoclonal anti DDIT3 (Cat. 179823; Abcam, Cambridge) was used at 1:200 dilution, and anti-parvalbumin (clone PARV-19; Cat. P3088, Sigma-Aldrich, St. Louis, MO) was used at 1:1000 dilution. For all antibodies, incubation with primary antibodies was performed overnight in a moist chamber. Immunostaining was performed using the EnVisionTM Dual Link (Cat. K4061; Dako, Hamburg, Germany). The samples were stained with hematoxylin and eosin and analyzed using a light microscope.

### 2.4. Statistical Analysis

Continuous variables were summarized using mean ± standard deviation, whereas categorical variables were summarized as frequency (%). Bivariate hypothesis tests were conducted using two-sided t-tests or Wilcoxon Rank Sum tests for continuous variables and chi-square or Fisher’s Exact tests for categorical variables. All statistical analyses were performed using SAS 9.4 (SAS Institute Inc., Cary, NC).

## 3. Results

There were 73 participants in the WTC cohort of responders who were eligible to be included in this study, four of whom had to be excluded as they did not speak English or because they had no viable contact information. Of the remaining 69 WTC thyroid cancer patients, 37 patients (54%) consented to participate. We were able to obtain FFPE thyroid tumor tissue samples for 30 WTC participants. The comparison of the 30 WTC thyroid cancer patients who consented and the 43 remaining WTC thyroid cancer patients showed that the groups were not significantly different for age at diagnosis, gender, and histology (Table 1).

**Figure 2:**
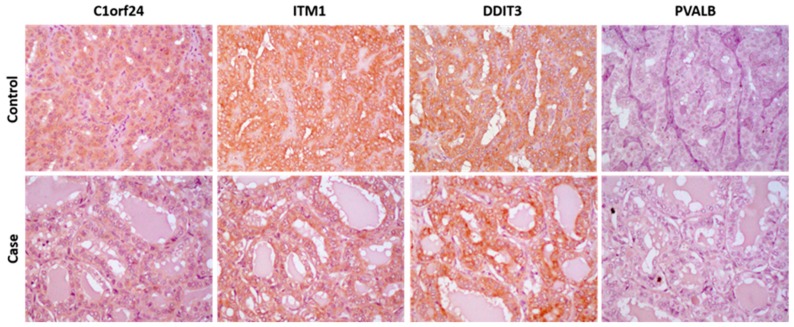
Representative results of molecular markers in WTC and non-WTC thyroid carcinomas (original magnification of ×40).

After matching, the WTC- and the non-WTC thyroid patient groups were well balanced in terms of age at diagnosis (*p* = 0.57), gender (*p* = 1.00), and histology (*p* = 1.00) (Table 1). Evaluation of clinical and pathological characteristics showed that there was no statistically significant difference in terms of tumor size (*p* = 0.77). Microcarcinomas, defined as thyroid cancer ≤1cm, were found in 52% of the WTC thyroid cancer patients compared with 40% in the non-WTC thyroid cancer patients (Table 1). Antibody assessment correctly classified thyroid nodules in either group (Figure 2, Supplementary Table S1).

Except for three thyroid samples, all thyroid tumor tissue samples from thyroid tissue from the two groups and all histology types tested positive for malignancy with antibodies C1orf24, ITM1, and DDIT3 and negative PVALB (Supplementary Table S1). For the other three samples (two WTC cases and one control), it was not possible to detect actual tumor tissue in the slides used for immunohistochemistry, and therefore, these samples could not be confirmed as malignant in the antibody-based test; all three samples were taken from tumors originally less than 0.3 cm in size. Most of the tumors had a strong brown staining for three markers (DDIT3, ITM1, and C1orf24) and no staining for the benign Hurthle adenoma marker (PVALB) (Figure 2; Supplementary Table S1).

## 4. Discussion

The findings of the present study suggest that overdiagnosis by virtue of misdiagnosis of a benign tumor as malignant does not explain the increased incidence of thyroid cancer observed in WTC responders. If overdiagnosis were occurring, we would expect an excess of false-positive malignancies among WTC thyroid cancer cases; benign tumors would be detected because of enhanced screening efforts and diagnosed as malignant because of physician bias associated with knowing that the patient has a history of WTC exposure. However, there was not an excess of false-positive thyroid cancer diagnoses found among the WTC thyroid cancer cases. In fact, none of the WTC thyroid cancer tumors assessed were false-positives; instead, all samples tested using the antibody-based cancer panel were determined to be true malignant disease. Although it may still be that physicians treating WTC responders may have biases that make them more likely to defensively diagnose nodules as thyroid cancer to avoid missing malignancies, our results suggest that screening of WTC responders, at least in the case of thyroid cancer, may not be unwarranted. 

It is important to note that surveillance bias may still be occurring in this cohort. Surveillance bias occurs when increased screening efforts result in nodules being detected that would otherwise have gone unnoticed given routine surveillance, thus inflating the actual incidence of disease in a heavily screened population. Smaller tumor size and younger age at diagnosis in the WTC cohort would generally be suggestive of surveillance bias, but the design of the present study does not allow any such conclusions to be drawn. For this study, WTC and non-WTC cases were matched by age, gender, and histology; thus, it is expected that age and tumor size are similar between the two groups. However, a descriptive study of the WTC thyroid cancer cases showed that these cases had similar clinical characteristics as thyroid cancer cases in the Mount Sinai registry in terms of age at diagnosis and tumor size, suggesting that surveillance bias alone cannot explain the excess risk of thyroid cancer in WTC responders [17].

Being that the increased risk of thyroid cancer in WTC responders does not appear to be an artifact due to physician bias, the results of this study leave open the possibility of an as-yet unknown carcinogenic mechanism through which WTC exposure is acting on thyroid cancer carcinogenesis. The biological basis of the tumors of WTC responders, including thyroid cancer tumors, warrants further research.

This is the first study to investigate the possible reasons for the increased incidence of thyroid cancer in the WTC population and the first to show that over-diagnosis due to physician bias does not appear to adequately explain the observed excess risk. The study is also novel in that it is the first to utilize biomarkers of malignancy in a WTC cohort. A further strength of this study is the high rate of compliance, with 54% of eligible WTC thyroid cancer patients agreeing to have their tumor sample molecularly analyzed.

This study is limited in that not all tumor samples could be retrieved from the institutions where the thyroid cancer surgery was performed and that, for a few samples, there was no tissue sample in the slides provided. However, this was a small portion of the samples (5%). In addition, a comparison of the characteristics of those patients for which we obtained a sample to the characteristics of the eligible patients does not show any difference in personal or tumor characteristics, thus reducing the possibility of selection bias.

Further analysis is needed to investigate a causal link between thyroid cancer in WTC-responders and high levels of exposure to potentially carcinogenic agents at Ground Zero, which may result in cancers with a shorter latency period, similar to that observed in individuals exposed to iodine-131 after the Chernobyl accident. It is well known that patients exposed to Chernobyl fallout demonstrated a linear dose–response association. Therefore, to define whether there is a correlation between the levels of exposure to the debris cloud and thyroid cancer, diagnosis is needed, as well as a longer follow-up. [18]

Additionally, the investigation of somatic events that drive thyroid cancer pathogenesis in this cohort, concomitant to longer follow-up and correlation with clinical-pathological features, will increase our knowledge of whether the molecular events in WTC responders differ from those not exposed to the dust cloud, as well as better define tumor aggressiveness [19,20].

## 5. Conclusions

In conclusion, rather than overdiagnosis of false-positives due to physician bias, it might instead be the case that the yearly screening visits by the WTCHP are identifying true cases of thyroid cancer earlier, increasing the possibility of a favorable prognosis, which warrants regular screening of this cohort.

## Figures and Tables

**Table 1:** Characteristics of the study sample.

Clinical–Pathological Features	WTC Thyroid Cancer Cases			Non-WTC Controls	
	Eligible (*n* = 43)	Included (*n* = 30)	*p* Value ^a^	Included (*n* = 30)	*p* Value ^b^
**Age at Diagnosis** (years)	48.5 (SD 7.7)	49.3 (SD 8.6)	0.65	47.8 (SD 11.3)	0.5652
**Gender**			0.16		1.00
Male	36 (83.7%)	21 (70%)		21 (70%)	
Female	7 (16.3%)	9 (30%)		9 (30%)	
**Histology**			0.74		1.00
Papillary thyroid carcinoma	27 (62.8%)	21 (70%)		21 (70%)	
Papillary thyroid carcinoma, follicular variant	12 (27.9%)	6 (20%)		6 (20%)	
Other	4 (9.3%)	3 (10%)		3 (10%)	
**Tumor Size**^c^ (cm)		1.38 (SD 1.16)		1.46 (SD 0.93)	0.78
**Microcarcinoma**					
**Yes** ^c^		14 (51.85%)		12 (40.0%)	0.37

^a^ Eligible versus included WTC thyroid cancer cases; ^b^ Included WTC versus non-WTC thyroid cancer cases; ^c^ Tumor size unknown for 3 WTC cases; WTC: World Trade Center.

## Supplementary Materials

The following are available online at https://www.mdpi.com/1660-4601/16/9/1600/s1, Table S1: Antibody-based test results and final histology in WTC cases and non-WTC cases.
