# Supplementary material for: Molecular Study of Thyroid Cancer in World Trade Center Responders
Source: Int J Environ Res Public Health. 2019 May 7;16(9):1600. doi: 10.3390/ijerph16091600 (PMC6539993; doi:10.3390/ijerph16091600)
Supplement: Supplementary file 1 [file ijerph-16-01600-s001.pdf]

Supplementary Table S1. Antibody-based test results and final histology in WTC cases and non-WTC cases.

Number	Case/ Control	C1orf24	ITM1	DDIT3	PVALB	Antibody-based results	Final Histology
1	control	+	+	+	-	Malignant	PTC
2	control	+	+	+	-	Malignant	PTC
3	control	+	+	+	-	Malignant	PTC
4	control	+	+	+	-	Malignant	PTC
5	control	+	+	+	-	Malignant	PTC
6	case	+	+	+	-	Malignant	PTC
7	case	No tissue	No tissue	No tissue	No tissue	No tissue	PTC
8	case	+	+	+	-	Malignant	PTC
9	case	ND-	ND-	ND-	ND-	No tumor in the slide	PTC
10	case	+	+	+	-	Malignant	PTC
11	control	+	+	+	+	Malignant	PTC
12	control	+	+	+	-	Malignant	FVPTC
13	control	ND-	ND-	ND-	ND-	No tumor in the slide	PTC
14	control	+	+	+	-	Malignant	PTC
15	control	+	+	+	-	Malignant	PTC
16	control	+	+	+	-	Malignant	PTC
17	control	+	+	+	-	Malignant	PTC
18	case	+	+	+	-	Malignant	PTC
19	case	+	+	+	-	Malignant	FVPTC
20	case	ND-	ND-	ND-	ND-	No tumor in the slide	PTC
21	case	+	+	+	-	Malignant	PTC
22	case	+	+	+	-	Malignant	PTC
23	case	+	+	+	-	Malignant	PTC
24	case	+	+	+	-	Malignant	PTC

25	case	+	+	+	-	Malignant	FVPTC
26	case	+	+	+	-	Malignant	FTC
27	case	+	+	+	-	Malignant	FVPTC
28	case	+	+	+	-	Malignant	PTC
29	case	+	+	+	-	Malignant	PTC
30	case	+	+	+	-	Malignant	FTC
31	control	+	+	+	-	Malignant	FVPTC
32	control	+	+	+	-	Malignant	FTC
33	control	+	+	+	-	Malignant	FVPTC
34	control	+	+	+	-	Malignant	PTC
35	control	+	+	+	-	Malignant	PTC
36	control	+	+	+	-	Malignant	FTC
37	case	+	+	+	-	Malignant	CCVPTC
38	control	+	+	+	-	Malignant	PTC
39	case	+	+	+	-	Malignant	PTC
40	control	-	+	+	-	Malignant	PTC
41	case	+	+	+	-	Malignant	PTC
42	control	+	+	+	-	Malignant	PTC
43	case	+	+	+	-	Malignant	PTC
44	control	+	+	+	-	Malignant	PTC
45	case	+	+	+	-	Malignant	FVPTC
46	control	+	+	+	-	Malignant	FVPTC
47	case	+	+	+	-	Malignant	PTC
48	control	+	+	+	-	Malignant	PTC
49	case	+	+	+	-	Malignant	PTC
50	control	+	+	+	-	Malignant	PTC
51	case	+	+	+	-	Malignant	FVPTC
52	control	+	+	+	-	Malignant	FVPTC

53	case	+	+	+	-	Malignant	HCC
54	control	+	+	+	-	Malignant	HCC
55	case	+	+	+	-	Malignant	FVPTC
56	control	+	+	+	-	Malignant	FVPTC
57	case	+	+	+	-	Malignant	PTC
58	control	+	+	+	-	Malignant	PTC
59	case	+	+	+	-	Malignant	CCVPTC
60	control	+	+	+	-	Malignant	PTC

PTC: papillary thyroid carcinoma; FVPTC: follicular variant of papillary thyroid carcinoma; CCVPTC: columnar cell variant of papillary thyroid carcinoma; FTC: follicular thyroid carcinoma; HCC: Hurthle cell carcinoma; ND: not determined.
